# An Investigation of the Usability of Alkali-Activated Blast Furnace Slag-Additive Construction Demolition Waste as Filling Material

**DOI:** 10.3390/ma18020398

**Published:** 2025-01-16

**Authors:** Talha Sarici, Tacettin Geckil, Bahadir Ok, Huseyin Suha Aksoy

**Affiliations:** 1 Department of Civil Engineering, Faculty of Engineering, Inonu University, 44280 Malatya, Türkiye; tacettin.geckil@inonu.edu.tr; 2 Department of Civil Engineering, Adana Alparslan Turkes Science and Technology University, 01250 Adana, Türkiye; bahadirok@atu.edu.tr; 3 Department of Civil Engineering, Engineering Faculty, Firat University, 23100 Elazig, Türkiye; saksoy@firat.edu.tr

**Keywords:** construction and demolition wastes, geopolymer, alkaline activation, blast furnace slag, soil stabilization, plate-loading test

## Abstract

In this study, the usability of construction and demolition waste (CDW) aggregates as filling when stabilized with alkaline activator solution (AAS) and blast furnace slag (BFS) was investigated. The initial stage of this study involved determining the engineering properties of CDW by laboratory experiments. In the next stage, modified Proctor tests were performed to investigate the compaction behavior of CDW, to which 5% to 30% BFS was added with water or AAS. In the following stage, California bearing ratio experiments were performed to determine the mixture specimen with the highest strength. In the final stage, a weak soil layer was created in a test tank, and fillings of different thicknesses were built on it using CDW with and without additives in the determined optimum mixing ratio. Then, plate-loading tests were conducted using a model foundation to evaluate the load–deformation behavior of the fillings. The study’s results indicated that adding BFS with water or AAS to CDW increased strength. Furthermore, the addition of 20% BFS yielded the highest strength value, and the CDW aggregates with the added BFS increased the ultimate bearing capacity by up to 4.72 times compared to those without the additive.

## 1. Introduction

The construction industry is responsible for the majority of natural resource consumption and waste generation globally [1,2]. Construction activities account for around 40% of carbon dioxide emissions from global energy consumption and consume around 40% of natural resources [2,3]. Natural aggregates from quarries are the most crucial natural resources utilized in engineering applications across various areas of the construction industry. Superstructures, infrastructure fillings in airports, highways, and railways, concrete production, and ground improvement techniques like stone columns, etc., all require natural aggregates. The construction industry is responsible for approximately 35% of the waste disposed of in landfills [1,2,3]. Consequently, in recent years, there has been a growing focus on researching the recycling of waste produced in the construction sector and exploring substitutes for depleted natural resources [4,5,6,7,8,9]. Construction and demolition waste (CDW) due to construction activities is vital to recycling, with an estimated production of approximately 1685 kg per person in the European Union in 2020 [6,10]. Additionally, CDW exhibits potential as a viable alternative to natural resources. The waste of concrete, brick, ceramics, tiles, asphalt, soils, metals, plastic, glass, plaster, wood, insulation materials, paper products, etc., can form CDW independently or in combination. CDW’s heterogeneous structure, CDW aggregates’ high water-absorption capacity, and their variable and low strength may restrict their recycling ratio [11,12,13,14,15].

Many studies have stated that CDWs can be an alternative to filling materials under foundations and transportation structures [16,17]. However, fillings created with CDW may cause problems in bearing capacity and settlement under load compared to fillings created with natural aggregates. Chemical additives, pozzolan additives, geosynthetics, etc., are used to eliminate these drawbacks and increase the performance of CDW. Investigating improvement methods for CDWs has recently become a popular research topic [13,18,19,20,21,22,23,24,25,26]. Del Rio Merino et al. [27] studied the existing practices employed to enhance the efficiency of recycling and reusing CDW. They highlighted that preventing the increase in CDW alone will not be enough, and therefore, CDW should be reused after recycling. Rahman et al. [24] conducted a study to examine the Young’s modulus and permanent deformation characteristics of CDWs. They achieved this by conducting repeated triaxial experiments on various types of CDWs, including recycled concrete aggregate, brick waste, and recycled asphalt pavement. In addition, the study also focused on improving the properties of CDWs. The study concluded that if their properties are enhanced, CDWs can potentially be a highly effective substitute for natural aggregates. Arulrajah et al. [22] stated that geopolymers produced by the alkali activation of industrial waste by-products can be advantageous additive materials for the environment, as they result in a much lower carbon footprint than cement. For this reason, the authors carried out a study on the usability of CDW in road base and subbase layers by stabilizing it with geopolymers. To improve the properties of CDW materials, they used calcium carbide residue (CCR), fly ash (FA), and slag (S) as additives in the geopolymers they produced. Moreover, they used a liquid alkaline activator containing sodium silicate solution and sodium hydroxide to activate their compositions. As a result of their study, they reported that CDWs stabilized with S showed significantly higher compressive strength than those stabilized with CCR and FA. Arulrajah et al. [23] investigated whether FA material is an alternative binder for CDW. They said that cement production causes serious carbon-dioxide emissions. Therefore, they stated that recent investigations have focused on alternative low-carbon cement binders. They stated that alternative binders such as FA significantly reduce environmental impacts and provide an economic advantage by reducing negative aspects such as storage. They conducted unconfined compression and cyclic triaxial loading tests to determine the effect of FA’s improvement on CDW. As a result of their tests, they reported that FA used with an alkaline activator had a significant improvement effect on CDW. They said that the CDW stabilized with 15% FA was the optimum mixture, but if more than 15% FA was added, there were unreacted FA grains, and therefore, only the fine content of the soil structure increased, which does not contribute to strengthening. Mohammadinia et al. [28] studied how to increase the strength of marginal and recycled materials without creating additional carbon emission sources by alkali activation of waste pozzolana products to stabilize CDW. They compared the stabilization strength results between high-calcium systems (such as blast furnace slag, BFS) and low-calcium systems (such as F-class FA). They reported that the high-calcium system was more efficient than the low one. They said that calcium maintains the alkalinity of the medium for the advancement of the activation of low-calcium waste particles and the formation of aluminosilicate gel for long-term strength development, as well as the early-stage strength development of C-A-S-H gel from the hydration process. Hasan et al. [29] examined the effect of blast furnace slag (BFS) and CDW on improving clay soil by performing unconfined compressive tests. According to the test results, they emphasized that the strength of the clay increased with the addition of BFS and CDW and that the strength increased with the curing time. Bassani et al. [20] investigated the impact of incorporating an alkaline-activated solution (AAS) made of sodium hydroxide and sodium silicate into CDW aggregates. They also assessed the potential application of the improved CDW in the base and subbase layers of roads. Their research concluded that the elastic modulus of samples made with pure AAS was considerably greater than that of samples made with diluted AAS and water alone.

According to the research conducted by Tuik [30], a total of 109.2 million tons of waste, 29.4 million tons of which was hazardous, was generated in manufacturing industry workplaces, mining operations, thermal power plants, industrial zones, and households in 2022. However, it was stated that until 2023, an average of 125 million tons of CDW was generated each year in Türkiye and this amount increased every year with urban transformation. About 90% of this was rubble such as concrete, bricks, and mortar, and the rest was made up of materials such as wood, metal, and plastic. However, a significant portion of this waste cannot be recycled or managed properly [31,32]. While CDW is a very important problem all over the world, it is of vital importance for Türkiye, as most of the country has active faults. Natural disasters in recent years in Türkiye have led to a significant increase in CDW. Located at the intersection of the Arabian, African, and Anatolian plates, Türkiye has experienced many earthquakes in the last century [33]. As a result of the earthquakes that occurred between 1927 and 2020, around 850 thousand civil engineering structures were severely damaged or destroyed [34]. In addition, more than 520,000 buildings were destroyed after an earthquake sequence (M_w_ = 7.7 and 7.6) centered in Kahramanmaraş on 6 February 2023 that affected 11 provinces (Osmaniye, Malatya, Adana, Adıyaman, Diyarbakir, Elazig, Gaziantep, Kahramanmaraş, Hatay, Kilis, and Sanliurfa). It was calculated that these earthquakes would generate about 350 to 580 million tons of CDW. This means that more CDW was generated in one day than in Europe or Türkiye in a year. It was also found that the distribution of waste generated as a result of these earthquakes was 1,453,015 tons of hazardous waste, 16,273,770 tons of soil and stone mixtures, 21,698,360 tons of bituminous mixtures and wood waste, 57,151,932 tons of mineral fraction waste, 37,747,073 tons of reinforced concrete waste, and 935,317 tons of scrap iron waste [35].

Literature studies have shown that the recycling and reuse of CDW is very important worldwide. Moreover, this reuse is feasible and can make a significant contribution to sustainability. In particular, Türkiye’s high earthquake potential and the high number of non-earthquake-resistant building stock makes the recycling of CDW in Türkiye more important. Considering this situation, in this study, the usability of CDW aggregates obtained from a non-earthquake-resistant building in the Osmaniye province, which was seriously affected by the Kahramanmaraş-centered earthquakes on 6 February 2023, was investigated as filling material. It was also seen in the literature that various additives were used to improve the performance of CDW, and in this case they usually used traditional standard laboratory tests such as the Proctor test, unconfined compression test, triaxial test, and modulus of elasticity test to determine how much improvement there was. However, it was thought that evaluating the performance of CDW improved with additives through model laboratory tests followed by field tests would be more appropriate to determine the improvement ratio. Nevertheless, studies involving model laboratory tests and investigating the performance of the filling under load, formed with CDW and improved with additives and/or geosynthetics, have been observed to be quite limited. Because of that, this study, which comprehensively investigates CDW improved by additives and/or geosynthetics using model laboratory tests, can contribute to the existing literature and provide a foundation for future field studies. The study first focused on the properties of aggregates produced by crushing CDW from a demolished building to obtain the desired grain size. It then looked into the effect of adding BFS on the strength of CDW. This evaluation was carried out by adding AAS or water to the mixture of CDW and BFS. The compaction tests and California bearing ratio tests were performed on samples prepared with non-additive CDW and BFS-CDW mixtures with or without the addition of AAS to determine the optimum BFS ratio. Also, model plate-loading tests were carried out on fillings constructed using non-additive CDW and CDW with additives at the optimum rate on weak soil. Depending on the results of model plate-loading tests, load-deformation behaviors were compared.

## 2. Materials

CDW was employed as a granular filling material in the tests, while cohesive (fine-grained) soil was used to form a weak subgrade. In addition, BFS was used as a pozzolan additive, sodium hydroxide (NaOH) served as an alkaline activator, and geotextile was utilized to separate the soil layers.

CDW, cohesive soil, BFS, NaOH, and the geotextile were obtained within the study’s scope. The first step was to determine the properties of these materials. Next, test samples were prepared by adding BFS to CDW at various rates (5%, 10%, 15%, 20%, 25%, and 30% by mass of CDW). The test samples were made with either water or AAS, and the difference between the two cases was examined. The prepared test samples underwent modified Proctor tests. Based on the resultant data, California bearing ratio (CBR) test samples were prepared and tested. Then, model plate-loading tests to determine load–deformation behavior were carried out on granular fill layers placed on the weak subgrade at different heights. CDW and additive-added CDW, which have the highest strength according to CBR tests, were used as the granular fill material. In these model plate-loading tests, the effect of a geotextile layer between the fill layer and the weak subgrade was also investigated. Finally, SEM (scanning electron microscope) images of the selected test samples were taken and interpreted.

### 2.1. Construction and Demolition Waste

**Figure 1:**
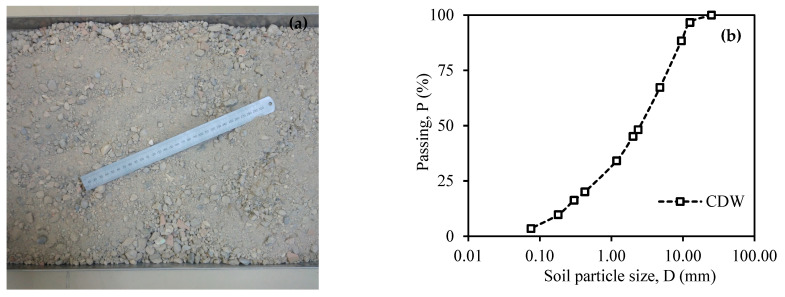
CDW aggregates: (**a**) image; (**b**) particle-size distribution curve.

The CDW used for this study was taken from a reinforced concrete building in Osmaniye, Türkiye. After examining its earthquake response, it was decided to demolish it as it was deemed inadequate. Unconfined pressure tests were performed on 24 core samples taken from the building to examine its earthquake performance. As a result of these tests, the average compressive strength value was determined to be 14.5 MPa [36]. Various metal pieces found in the debris resulting from the building’s demolition were picked over, and then the debris was crushed with a crusher. After the crushing process, CDW aggregates with grain diameters in the 0–20 mm range were formed for use in experiments (Figure 1a). The CDW aggregates were sieved according to the ASTM C136-06 [37] standard. Figure 1b presents the granulometry curve. The sieve analysis determined the soil class of CDW as SW (well-graded gravelly sand) by UCSC [38].

CDW included a wide variety of heterogeneous materials. Testing was conducted according to the BS EN 933-3 [39] standard to determine the mass fractions of various substances contained in CDW. Table 1 lists the mass ratios of different materials found in CDW. Considering Table 1, the following comments can be made regarding the chemical composition of CDW. Calcium (Ca) is present due to limestone-based materials such as concrete and mortar. In addition, concrete and concrete-based materials often contain calcium silicate hydrate (C-S-H), calcium hydroxide, and other calcium-based compounds. Silica (SiO_2_) is found in silica-based materials such as sand, bricks, and cement. Also, iron oxide (Fe_2_O_3_) is a component in cement and bricks. Furthermore, alumina (Al_2_O_3_) is present in bricks, ceramics, and cement. Additionally, wood in CDW contains lignin and cellulose, while plastics in CDW usually contain polymers. However, organic substances may be present in insulation materials, paints, and adhesives in CDW. Iron (Fe), aluminum (Al), and copper (Cu) are found in rebar, roofing, pipes, and electrical cables in CDW.

**Figure 2:**
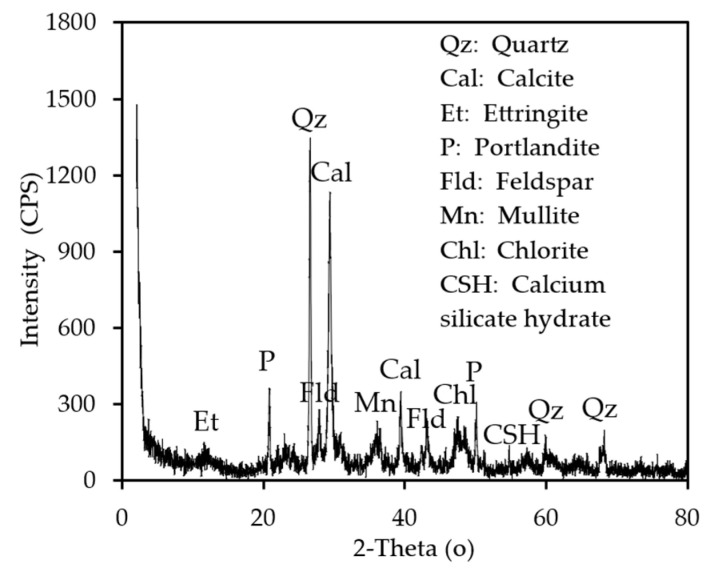
XRD patterns of CDW.

XRD (X-ray diffraction) analysis was performed to examine the crystal structures of CDW and to interpret the substances in it. Rigaku RadB-Dmax II and Rigaku RINT-2000 (Tokyo, Japan) X-ray diffractometer were used for the XRD analysis and Jade 6+ crystal analysis program and library integrated into these systems. According to Figure 2, there is evidence of quartz due to natural aggregates, sand, etc.; calcite due to cement mortar, lime-based binders, etc.; portlandite due to cement hydration products; feldspar due to ceramics, bricks, natural stone, etc.; mullite due to bricks, ceramics, etc.; ettringite and C-S-H due to cement hydration, etc.; and hematite due to materials containing ceramics, iron, etc. Since the CDW in this study contains relatively more natural aggregate and cement, it can be said that the content of quartz and calcite is higher.

A group of tests were performed on the CDW to determine its other physical and mechanical properties. The engineering properties of CDW are summarized in Table 2.

**Figure 3:**
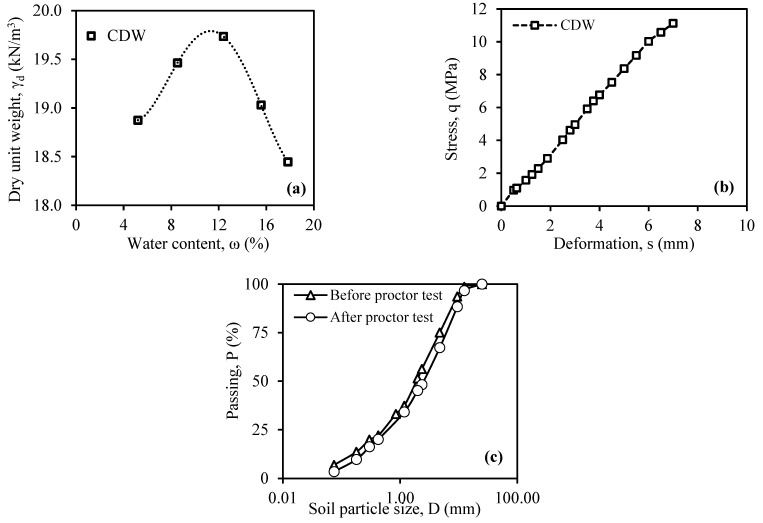
(**a**) Compaction curve; (**b**) stress–strain behavior in the CBR test; (**c**) gradation change before and after the modified Proctor test.

Following the modified Proctor test on CDW, Figure 3a displays the compaction curve. Figure 3b shows the stress–strain curve of CDW with optimum water content formed in the CBR tests. The CDW was put through sieve analysis after the modified Proctor tests to see how the material’s gradation changed (how it was crushed) compared to the results of the sieve analysis before the modified Proctor tests. Figure 3c shows the sieve analysis results before and after the modified Proctor test.

### 2.2. Cohesive (Fine-Grained) Soil

**Figure 4:**
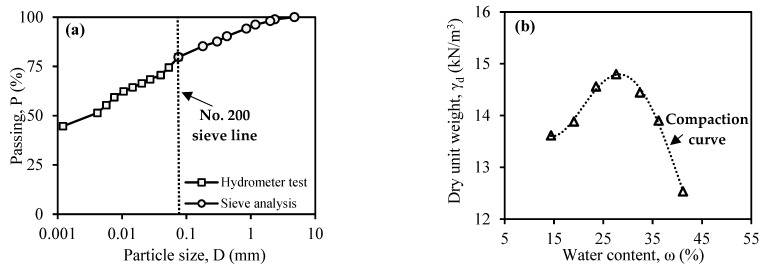
Curves of fine-grained soil: (**a**) gradation; (**b**) compaction.

In the model plate-loading test, a layer of CDW was placed on weak soil to examine the fill layer’s behavior under the most unfavorable conditions. The weak soil condition was created using cohesive (fine-grained) soil obtained from Malatya, Türkiye. A series of conventional geotechnical laboratory tests were carried out to determine the engineering properties of the supplied fine-grained soil. Table 3 summarizes the values obtained from all tests conducted on fine-grained soil. Figure 4a displays the gradation curve of cohesive soil. Based on the analysis of the gradation distribution and consistency limits, it has been concluded that the soil falls under the MH type (high plasticity silt) according to the ASTM D2487-11 [38] standard. Furthermore, Figure 4b displays the compaction curve derived from the Proctor test of the soil.

**Figure 5:**
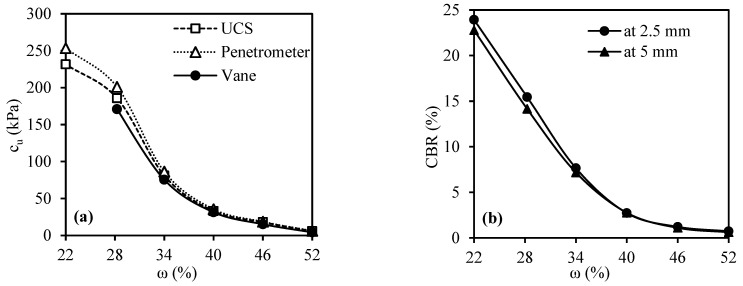
The strength of fine-grained soil based on water content variation: (**a**) c_u_ (**b**) CBR.

Various water contents (22%, 28.25%, 34%, 40%, 46%, and 52%) were tested on fine-grained soil using unconfined compression and CBR tests to determine the water content that could lead to weak soil conditions. Various instruments, including the vane and penetrometer, were used to determine fine-grained soil’s undrained shear strength value (c_u_) for different water contents. The c_u_ values obtained for different water contents due to unconfined compression (UCS), vane, and penetrometer tests are shown in Figure 5a. Additionally, CBR values obtained at different water contents are presented in Figure 5b. Based on the results in Figure 5, it was concluded that a water content of 46% is required for the cohesive soil to create a weak soil condition.

### 2.3. Blast Furnace Slag

In this study, blast furnace slag (BFS) was used as a pozzolan additive to improve the engineering properties of CDW. BFS was obtained from Karabük, Türkiye. The BFS grains were passed through sieve no. 200. BFS’s specific surface is 3996 cm^2^/g, and its unit weight is 28.66 kN/m^3^. The chemical composition of BFS was determined using the X-ray Fluorescence (XRF) technique available at Inonu University Scientific and Technological Research Center (Table 4).

### 2.4. Alkaline Activator Solution

This study used sodium hydroxide (NaOH) as an alkaline activator solution (AAS) to increase the strength values of CDW with or without added BFS. Thus, the aim was to create a geopolymerization reaction using BFS and NaOH. The experimental studies involved preparing AAS using solid NaOH and water. Table 5 displays the properties of NaOH. Within the scope of the study, 320 g of NaOH was used in solid form in 1 L of solution to prepare AAS, in other words, 8 molar NaOH solution was prepared. AAS was prepared considering the studies in the literature [21,22,23,24]. The use of NaOH in geopolymer production is an issue that can be subjected to a complex moral evaluation with both positive and negative aspects. Positive aspects include sustainability, waste management, and durability, while negative aspects include environmental impacts and cost. In conclusion, the ethical assessment of the use of sodium hydroxide in geopolymer production requires a multifaceted cost–benefit analysis.

### 2.5. Geotextile

A geotextile was used to separate the weak soil and the fill layer, and its impact on the bearing capacity was assessed based on the test results in the model plate-loading test. The geotextile was supplied in roll form and was cut into a circular shape to fit the inner diameter of the test tank. The properties of the geotextile used in the tests are presented in Table 6.

## 3. Methods

### 3.1. Modified Proctor and CBR Tests

Test samples for modified Proctor and CBR tests were prepared using BFS at varying percentages of CDW mass (0%, 5%, 10%, 15%, 20%, 25%, and 30%) with either water or AAS. This was carried out to observe any changes or improvements in the properties of the test samples due to the presence of BFS and AAS. The aim was to determine the optimum percentage of BFS required to achieve the desired test results. The CBR tests focused on measuring the samples’ strength and load–displacement behavior.

The ASTM D1557-12 [43] standard guided the conduct of experiments for modified Proctor testing. The optimum water content for samples created by combining CDW, mixing water, and BFS was expressed as ω_opt_. In experiments involving AAS, the optimum solution content was specified as ω_optAAS_. Within the scope of the study, 14 modified Proctor experiments were performed. The CBR test samples were prepared based on the ω_opt_ or ω_optAAS_ values obtained from modified Proctor experiments. Each test sample had its γ_dmax_ value. The CBR tests were carried out following the guidelines of the ASTM D1883-14 [44] standard. In this study, 84 CBR tests were performed. In CBR tests, experiments were repeated 3 times for each sample to reduce the experimental error. Mean values were given when presenting the CBR test results.

### 3.2. Model Plate-Loading Test

**Figure 6:**
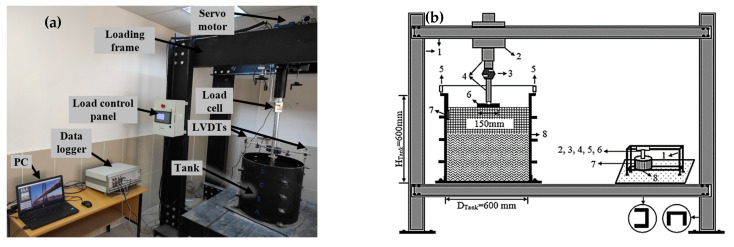
Model plate-loading test system: (**a**) image; (**b**) drawing.

The experimental system was designed to conduct complex tests on weak soil and the fill material placed on it. This system consisted of multiple components, including a data logger that collected data from all the sensors installed throughout the setup. The data logger was connected to a computer that served as the central hub for the data. The computer displayed and recorded all the data collected by the data logger. The model plate-loading test system is presented in Figure 6a. The experimental setup comprises the following components, as shown in Figure 6b:

1. The frame is constructed of rigid steel profiles.
2. The loading engine (servo motor) is located on top of the frame.
3. A load cell records the load data during the test.
4. The loading piston transfers the servo motor’s movement to the model foundation.
5. Linear variable differential transformer (LVDT) sensors measure displacement values.
6. The model foundation is where the plate is positioned for testing.
7. A pressure meter is used to measure the pressure on the tank’s inner surface.
8. The test tank.

The experimental system included various pieces of compression equipment. These pieces of equipment were used to place the weak soil and fill material into the test tank. The compression equipment was designed to work with the other system components to ensure that the tests were carried out accurately and efficiently.

The cohesive soil and CDW materials were dried in an oven. The cohesive soil was then mixed with water to achieve a water content of 46%. After thorough mixing, the soil was kept at room temperature and at high humidity for one day to maintain its water content. The water content was checked before plate-loading tests were conducted. The CDW was also mixed with water to achieve the optimum water content determined by the modified Proctor test. The mixture was then kept at room temperature and in a humid environment for a day to avoid losing its water content. To prevent the sample from hardening, BFS and CDW were mixed in dry form about two hours before the experiment. AAS was then added to this dry mixture, thoroughly mixed, and left for approximately two hours. Samples of the waiting mixture were taken to check the water content and ensure that the experiment was conducted under suitable conditions.

The cohesive soil was placed in the tank in 5 cm layers. The targeted water content of the cohesive soil is 46%. In addition, its natural unit volume weight is 17.45 kN/m^3^, its undrained shear strength is 18 kPa, and its CBR value is 1.2%. Mass control, water content, and strength control were performed for each cohesive soil layer. First, the wet cohesive soil was weighed, and the required soil amount for each layer was adjusted (mass control). Then, water content samples (water content control) were taken from different soil points allocated for each layer before the experiment. After these procedures, the soil at the mass calculated for one layer was compacted in the tank. The targeted unit volume weight was achieved by controlling the compacted soil layer’s thickness. After the compression process was completed, vane and penetrometer tests (strength control) were carried out at various points on the upper surface of the layer. These processes were repeated, and the cohesive soil was placed in the rigid test tank in layers in a controlled manner to the desired height. In all test series, the total height of the weak soil layer was 350 mm.

Fill layers on the cohesive soil with the weak soil condition were created using CDW with optimum water content or AAS+BFS-added CDW prepared in optimum liquid content. These materials were compacted into the rigid test tank in 5 cm layers to ensure the maximum dry unit volume weight value. While creating the layers, mass and water content were checked for each layer. Model plate-loading tests were carried out by placing geotextile between the weak soil and the fill layer, both to prevent the fill material and weak soil from mixing and to examine the effect of the presence of geotextile on the bearing capacity.

A circular model foundation with a diameter of 150 mm was placed in the middle of a rigid test tank after creating the desired test section. The model plate-loading tests were carried out with a loading rate of 1 mm/min in all the tests. A pressure gauge was put on the edge of the test tank to check if a boundary effect had been created. The stress values measured during the experiments via a pressure gauge were shallow. The maximum stress value measured was approximately 0.6% of the stress value occurring during loading. Hence, it was concluded that the boundary effect was negligible.

**Figure 7:**
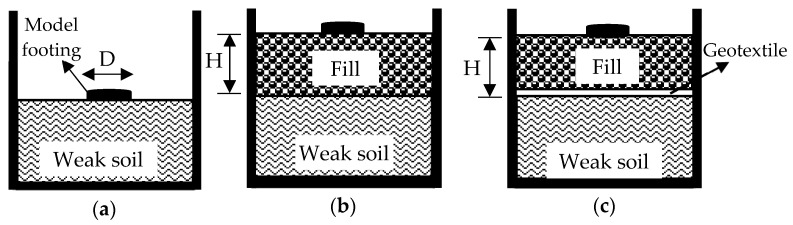
Model plate-loading tests: (**a**) Series I; (**b**) Series II; (**c**) Series III and IV.

The model plate-loading tests were carried out under controlled room temperature conditions. These tests were conducted at the end of a 7-day cure, ensuring that there was no loss of moisture in the test section that was produced. The experimental study programmed for model plate-loading tests carried out in the framework of this research is shown in Table 7. The tests were planned in four series: I, II, III, and IV. Series I involved testing weak soil. Series II comprised testing the CDW fill, constructed at various heights on the weak soil. Series III involved testing the geotextile to separate the weak soil and CDW fills built at different heights. Finally, Series IV consisted of testing CDW fills with added BFS and AAS. Figure 7 summarizes all the test series.

The plate-loading tests were conducted to determine the bearing capacity of the model plate. The results were presented in the form of pressure (q)–displacement ratio (s/D) curves, where the displacement ratio was calculated by dividing the resulting displacement by the model plate diameter. However, the curves did not provide significant collapse results. Therefore, the 0.1B method, which is both practical and objective, was used to determine the ultimate bearing capacity value (q_ult_). This method has been recommended by experts such as Briaud and Jeanjean [49] and Örnek [50].

### 3.3. Scanning Electron Microscope and Energy Dispersive X-Ray Spectroscopy

Scanning electron microscopy (SEM) and energy dispersive X-ray spectroscopy (EDS) analyses were performed to observe the internal structures of the test samples prepared within the scope of the study through the images obtained and to determine the elements formed as a result of reactions in the test samples. Thus, we tried to interpret how the strength of CDW was increased by additives, especially by determining whether the geopolymer reaction occurred. The formation of reaction products such as aluminosilicate gel and calcium-rich phases were analyzed by SEM-EDS. These gels are key components that increase the strength of geopolymers. Furthermore, voids in the matrix and the microstructure homogeneity of the test specimens were analyzed by SEM images. Properties such as reduced voids and increased microstructure homogeneity can have an improvement effect on strength. EDS was used to analyze the distribution of elements in the test samples, especially the presence of elements such as silicon (Si), aluminum (Al), calcium (Ca) and sodium (Na). In summary, SEM was used to visualize the formation process of the matrix of the test specimens, while EDS was used to quantitatively analyze the chemical compositions and determine the reaction mechanism.

The SEM-EDS analyses for this study were carried out at the Inonu University Scientific and Technological Research Center. SEM analyses were performed on the LEO-EVO 40 test setup and images were obtained from the surfaces of the materials using the secondary electron (SE) and backscatter electron detector (BSD). Bruker-125 eV (Billerica, MA, USA) was used in the EDX test setup. To improve the quality of the images obtained from the samples before SEM-EDS analysis, the sample surface underwent a coating process using gold–palladium powders. SEM-EDS analyses were carried out on the test samples with the optimal pozzolana additive material ratios (20% BFS and 20% BFS+AAS) as determined by CBR tests, as well as from the CDW and CDW+AAS test samples. Additionally, SEM-EDS analysis was also performed on the powdered form of BFS.

## 4. Results

### 4.1. Compression of the Samples

**Figure 8:**
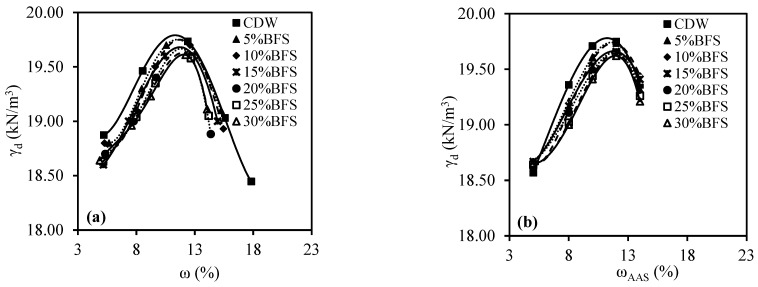
The compaction curves of BFS-added CDW: (**a**) with water; (**b**) with AAS.

Figure 8 displays the compaction curves of CDW and BFS-added CDW at various percentages. It shows the water content (ω)-dry unit volume weight (γ_d_) (Figure 8a) and the AAS content (ω_AAS_)-γ_d_ (Figure 8b) curves. During the modified Proctor tests conducted on CDW samples with the addition of BFS and water, it was recorded that the ω_opt_ value increased as the BFS ratio increased, while the γ_dmax_ value decreased. Similarly, during the modified Proctor tests on CDW samples with the addition of BFS and AAS, it was determined that the ω_optAAS_ ratio generally increased as the BFS ratio increased, while the γ_dmax_ value decreased. The increase in optimum liquid content values was attributed to BFS’s high water absorption potential, which stems from its high CaO content. Meanwhile, the decrease in γ_dmax_ value was linked to the maximum BFS grain diameter of 75 microns, resulting in reduced mass due to its relatively lighter weight. The Proctor curves produced in both water and AAS exhibited distinct behaviors, but the optimum liquid ratio (ω_opt_ and ω_optAAS_) and γ_dmax_ values were similar. This result was thought to be due to AAS’ ability to reduce friction between grains (possibly due to its lubricant structure).

### 4.2. Evaluation of CBR Tests’ Results

**Figure 9:**
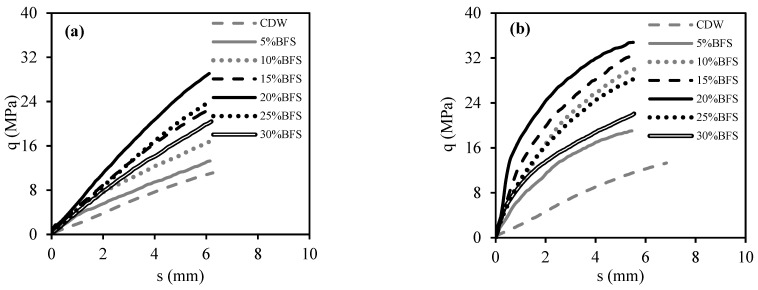
CBR tests’ results of CDW samples with BFS: (**a**) cured for 7 days; (**b**) cured for 28 days.

**Figure 10:**
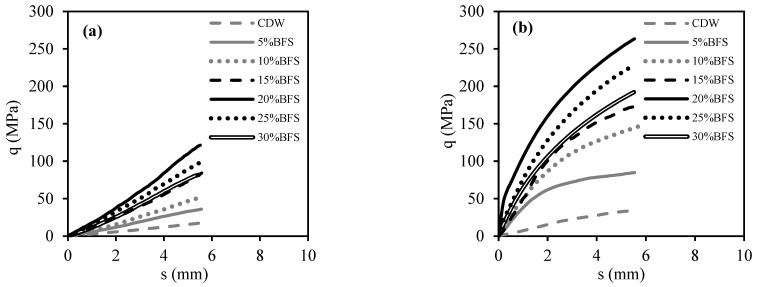
CBR tests’ results of CDW samples with BFS and AAS: (**a**) cured for 7 days; (**b**) cured for 28 days.

Figure 9 shows the average stress (q)–displacement (s) curves from CBR tests carried out with CDW samples that had been cured for 7 and 28 days with different amounts of BFS added (5% to 30%). Figure 10, on the other hand, displays the q–s curves of CDW samples with BFS+AAS added, which were also cured for 7 and 28 days. All CBR tests observed that the BFS additive increased the CDW’s CBR value, while 20% of the BFS additive created the highest CBR value. According to Figure 9 and Figure 10, curing the test samples for 28 days caused an increase in the initial slope of the q–s curves, which means more rigid behavior.

As seen in Figure 9, it was determined that the strength increased as the BFS increased up to 20%, the effect of improving the strength peaked at 20%, and the addition of BFS after 20% tended to decrease the strength. The main reason for this behavior was that the BFS remained unreacted after 20% additive content increased the fine grain content of CDW. Because the water content of the test specimens was determined by a modified Proctor test to obtain the highest density, increasing the water content can encourage unreacted BFSs to react, but reduces the density value of the filling. This is undesirable, as dry density is a measure of compaction. In addition, increasing the curing time significantly increased both strength and rigidity. This shows that the reaction does not end within 7 days and continues up to 28 days [21,22,23].

As in the usage of BFS with water, it was determined from Figure 10 that the addition of more than 20% BFS also formed unreacted BFS and therefore did not increase the strength after this ratio. Again, it was found that the curing time significantly increased the strength and rigidity. Comparing Figure 9 and Figure 10 to observe the effect of the usage of AAS, it was found that the usage of AAS significantly increased the strength and rigidity for all curing times, as it significantly induced the geopolymer reaction. This was confirmed by the SEM-EDS analyses given in the following chapters. AAS increases the dissolution of aluminosilicate-based raw materials such as BFS. In particular, they ensure the dissolution of silicon and aluminum ions and accelerate the start of the reaction. High pH activators such as NaOH accelerate the reaction by providing more ion solubility. The appropriate amount and chemical structure of AAS leads to the formation of a dense and homogeneous geopolymer gel, which improves the mechanical strength. This was also clearly seen in test samples containing AAS and BFS. AAS strengthens the binding forces in the aluminosilicate matrix. This has a direct impact on the curing process and ultimate durability of the geopolymer [14,19,20].

A bearing capacity ratio recommended by Binquet and Lee in 1975 [51] was utilized to evaluate the results obtained from the CBR tests. Hence, Equation (1) was employed to determine the bearing capacity ratio, providing a reliable measure of the soil’s load-bearing capacity.

(1) $${BCR}_{CBR}=\frac{{CBR}_{u}}{{CBR}_{o}}$$

where *CBR_u_* is the CBR value of the test for which the BCR_CBR_ value needs to be calculated; and *CBR_o_* is the CBR value of the CDW test sample prepared with water at the end of the relevant curing period.

**Figure 11:**
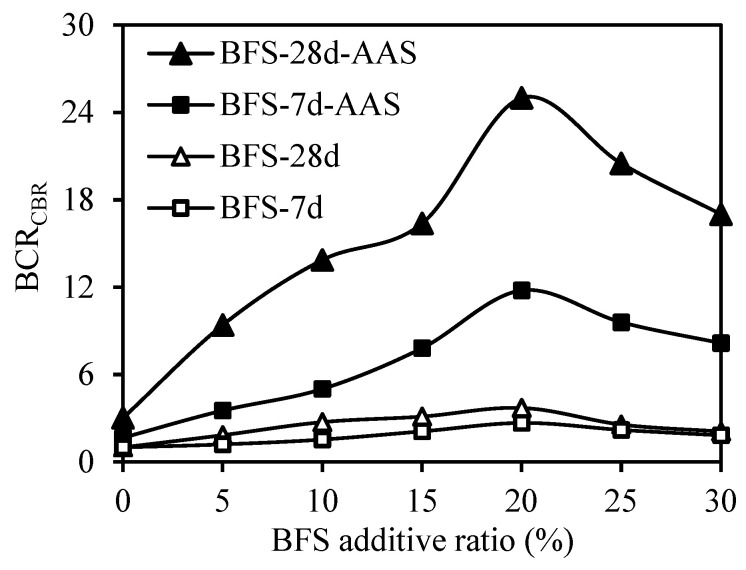
Effect of BFS additive ratio on BCR_CBR._

In Figure 11, the BCR_CBR_ values obtained from CBR tests are presented. The results indicate that curing time had a positive effect on the strength in all tested series. Moreover, the inclusion of BFS and AAS additives resulted in a further increase in strength. The most significant improvement was observed when a 20% BFS additive was used. The CBR value of the CDW sample with 20% BFS added exhibited an increase of approximately 3 times compared to the unmodified CDW sample for both 7-day and 28-day curing periods. Upon the addition of AAS to the CDW sample with 20% BFS, the CBR value showcased a substantial improvement, showing an approximately 12 and 25 times increase compared to the unmodified CDW sample for the 7-day and 28-day curing times, respectively.

### 4.3. The Assessment of Model Plate-Loading Tests

**Figure 12:**
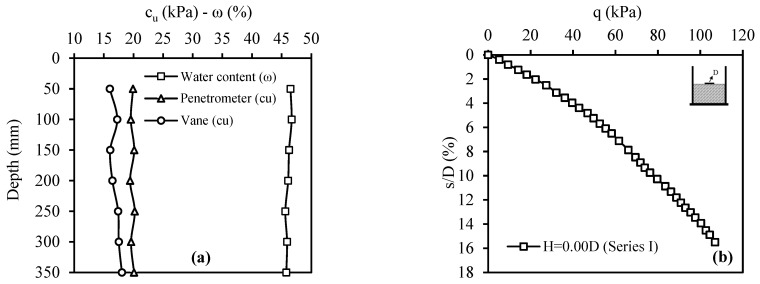
Series I: (**a**) water content and strength values along the soil depth; (**b**) q–s/D curve.

The bearing capacity of the weak soil was determined with the model plate-loading test (Series I). Figure 12a gives the strength and water content of the weak soil layers determined by penetrometer, vane, and water content tests from the surface to the depth. Moreover, Figure 12b presents the weak soil’s stress (q)–displacement ratio (ratio of displacement to model foundation diameter) (s/D) curve.

**Figure 13:**
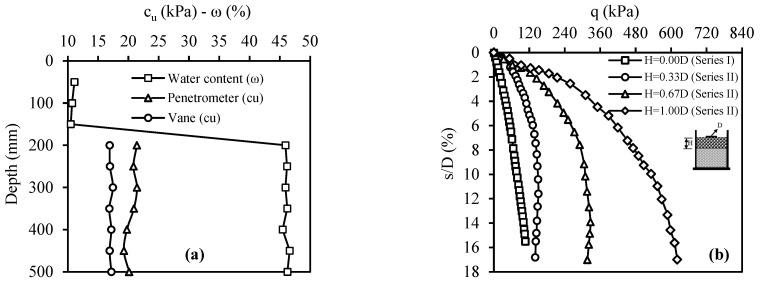
Series II: (**a**) water content and strength values along the soil depth for test number 4; (**b**) q–s/D curves.

Model plate-loading tests involved placing fill layers formed with CDW in three different thicknesses, H/D = 0.33, 0.67, and 1.00, on the weak soil (the fill thickness and plate diameter are represented by H and D, respectively). The primary focus of the Series II tests was on understanding the effect of fill layer thickness. In the case of the test number 4, the strength values of the weak soil and the water contents of both the weak soil and the filling layer along the depth are presented in Figure 13a. The q–s/D curves obtained from the Series II tests are shown in Figure 13b. The key finding was that the CDW fill layer consistently enhanced the bearing capacity of weak soil in all cases. Moreover, as the fill layer thickness increased, the bearing capacity also increased. This trend was attributed to the fact that the CDW grains remaining on the slip surfaces formed by the load effect on the plate exhibited better resistance than the weak soil. Importantly, this resistance was found to increase proportionally with the CDW thickness. In other words, placing a rigid fill layer on weak soil increases the bearing capacity by spreading the applied load over a larger area, thus reducing the stress per unit area.

**Figure 14:**
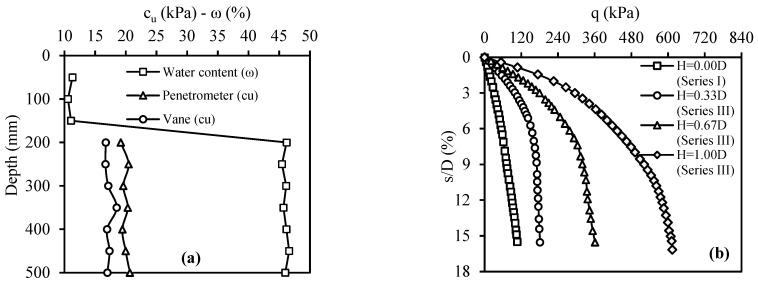
Series III: (**a**) water content and strength values along the soil depth for test number 7; (**b**) q–s/D curves.

The tests in Series III investigated the impact of using geotextiles to separate the fill layer and weak soil interface on bearing capacity. In this Series, model plate-loading tests were performed on CDW fill layers with three different thicknesses: H/D = 0.33, 0.67, and 1.00. In the case of test number 7, the strength values of the weak soil and the water contents of both the weak soil and the filling layer along the depth are presented in Figure 14a. Figure 14b displays the q–s/D curves obtained from the Series III tests. The results indicated that using geotextiles at the interface of the fill layer and weak soil enhanced bearing capacity values in all cases due to the membrane effect on the geotextile after loading.

**Figure 15:**
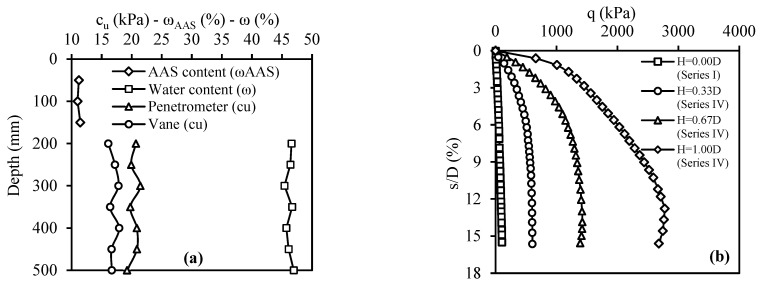
Series IV: (**a**) water content and strength values along the soil depth for test number 10; (**b**) q–s/D curves.

The Series IV tests aimed to evaluate the bearing capacity of CDW fill layers to which BFS-AAS was added, placed on weak soil. The CDW fill layers with three different thicknesses: H/D = 0.33, 0.67, and 1.00, were prepared by adding 20% BFS and AAS because this particular mixture showed the best performance among the others tested based on the results of CBR tests. In addition, in these tests, the geotextile was placed at the interface of the fill layer and weak soil. In the case of test number 10, the strength values of the weak soil and the water contents of both the weak soil and the filling layer along the depth are presented in Figure 15a. The q–s/D curves obtained from Series IV tests are shown in Figure 15b. The results showed that adding BFS-AAS significantly improved the bearing capacity in all cases, with greater improvement as fill layer thickness increased.

The bearing capacity ratio (BCR), as recommended by Binquet and Lee [51], was determined based on the findings of model plate-loading tests (Equation (2)). This approach was used to assess the specific contributions of geotextile and additive materials to the bearing capacity of the fill layer on weak soil.

(2) $${BCR}_{MPL}=\frac{q_{ur}}{q_{uo}}$$

where “*q_ur_*” is the q_ult_ value obtained from the model plate-loading tests from which the BCR_MPL_ value needs to be calculated; “*q_uo_*” is the q_ult_ value obtained from the model plate-loading test conducted on the weak soil.

**Figure 16:**
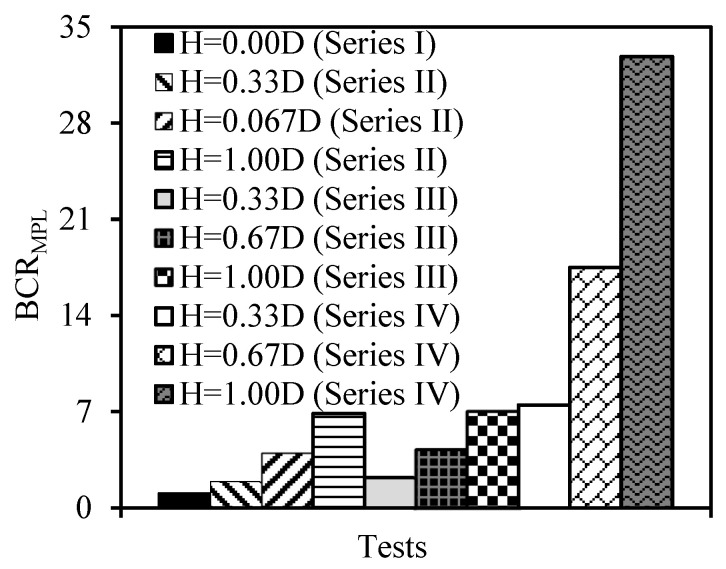
The BCR_MPL_ values obtained from the results of the model plate-loading tests.

Figure 16 presents the BCR_MPL_ values calculated from all model plate-loading tests. The figure indicates that various fill layers enhanced the weak soil’s bearing capacity. However, the geotextile had minimal impact on the bearing capacity, whereas the BFS-AAS significantly increased it. This enhancement could be attributed to the geopolymerization reaction that occurs between BFS, AAS, and CDW, resulting in a more rigid filler that is more resistant to shear failure.

### 4.4. The Assessment of SEM-EDS Analyses of the Samples

**Figure 17:**
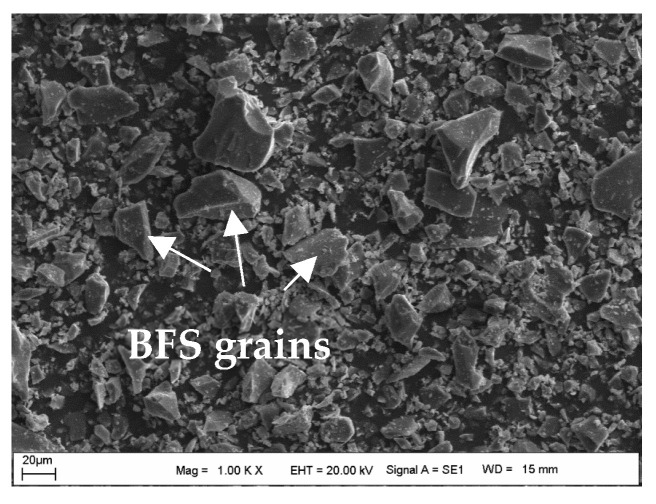
The SEM images of the BFS sample.

**Figure 18:**
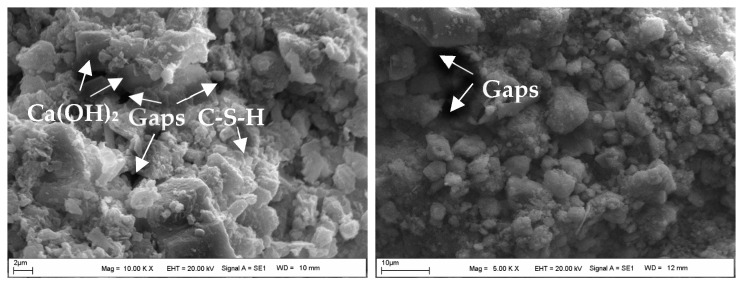
The SEM images of the CDW sample.

**Figure 19:**
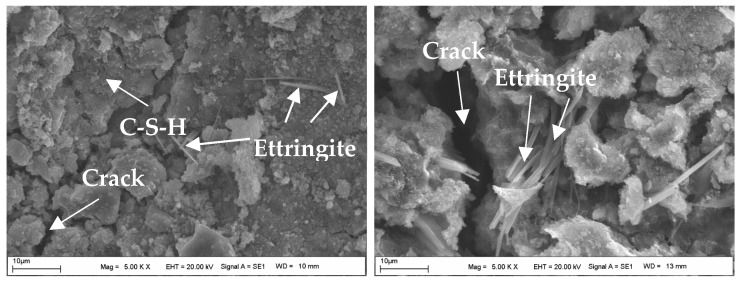
The SEM images of the CDW+AAS sample.

**Figure 20:**
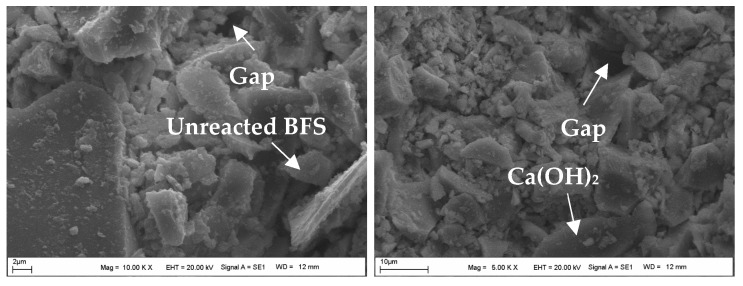
The SEM images of the CDW+20%BFS sample.

**Figure 21:**
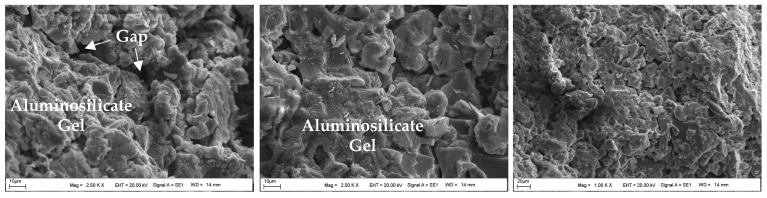
The SEM images of the CDW+20%BFS+AAS sample.

Figure 17 shows the SEM image of the powdered form of BFS, while the SEM images of the CDW test sample prepared with water are shown in Figure 18. Additionally, Figure 19, Figure 20 and Figure 21 display SEM images of samples of CDW+AAS, CDW+20%BFS, and CDW+20%BFS+AAS, respectively.

The SEM images revealed that BFS is composed of irregular and angular particles. This finding is consistent with previous studies [52,53]. On the other hand, SEM images of the CDW sample indicated the presence of small amounts of CSH and Ca(OH)_2_ phases in the microstructure, along with numerous micro-cavities. Interestingly, the microstructure of the CDW-AAS sample appeared to be more compact than that of the CDW sample, with the presence of ettringite crystals. This suggests that the strength of CDW increases only when AAS is added. Upon thorough analysis of the microstructure of the CDW+20% BFS sample, it is evident that a dense gel has formed within the matrix. Furthermore, a few unreacted BFS grains and Ca(OH)_2_ were observed. In contrast, the SEM observations of the CDW+20%BFS+AAS sample revealed a highly compact and void-free microstructure. The presence of dense aluminosilicate gels in the microstructure of the CDW+20%BFS+AAS sample unequivocally indicates a strong correlation between strength and microstructure. This correlation is attributed to the rapid geopolymerization rate of BFS and the high CaO content in laboratory conditions. Moreover, a study by Hardjito [54] underscores that raw materials with high CaO content can expedite the geopolymerization reaction, leading to increased strength and faster setting time. The SEM images vividly demonstrate that the gel structure formed due to the reaction of BFS with AAS, which effectively acted as a filling material to fill the voids in the CDW sample [55].

Additional information on the elemental composition of the test specimens within the scope of the study was obtained from EDS. The results obtained from EDS are summarized in Table 8. EDS results confirmed that BFS contains typical components (Ca, Si, Al, Mg, etc.) and minor elements (Mn, S, Ti, Fe, K, Na). This means that it does not show sufficient hydraulic activity alone but can form a strong binding phase with appropriate AAS or additives such as cement, etc. When the EDS analysis on CDW was examined, the higher content of calcium, silicon, and oxygen is due to the basic matrix in the concrete, while the partial iron content is due to rebar steel residue or aggregate containing iron oxide. Also, the presence of carbon was thought to be from carbonated cement, Ca(OH)_2_, or CaO reacted with CO_2_. The high Ca and O content (probably calcium carbonate/hydrate), medium levels of Si, and traces of iron supplemented by small amounts of Al and Mg confirm that this material is a concrete-mortar-based construction demolition waste. The presence of carbon (C) is also due to carbonation of the concrete. When the results obtained from CDW+AAS are examined, one remarkable fact is the combination of Ca-Si-Al-Na-Mg, which indicates the presence of C-A-S-H and/or N-A-S-H gel phases that can be formed by alkaline activation. This indicates that it is possible to develop a green binder (geopolymer) derived from CDW. This is because there is high calcium content in the mixture due to the concrete/mortar residues in the CDW. This calcium is dissolved in the AAS and re-incorporated into the binder structure and helps the formation of gels. EDS analyses for CDW+20%BFS showed that BFS did not participate in a substantial chemical reaction due to the absence of AAS; therefore, a strong binder phase did not develop. The presence of elements such as Mn, Mg, Ti, Fe, etc., reflects the BFS characteristic, while Ca and Si are of both BFS and CDW origin. This can lead to a mostly inefficient matrix due to the lack of alkaline environment. BFS largely acts as a filler, as it is not sufficiently reactive in the absence of alkali. The results of the EDS experiments for CDW+20%BFS+AAS showed that the expected geopolymerization reaction occurred. The high Ca/Si ratio and the presence of Al, Mg, Mn, S, etc. indicate that multicomponent phases can form in the gel. These phases contributed positively to the mechanical and durability properties of CDW.

### 4.5. A Comparison of the Results of the Tests with Those of Previous Studies

**Figure 22:**
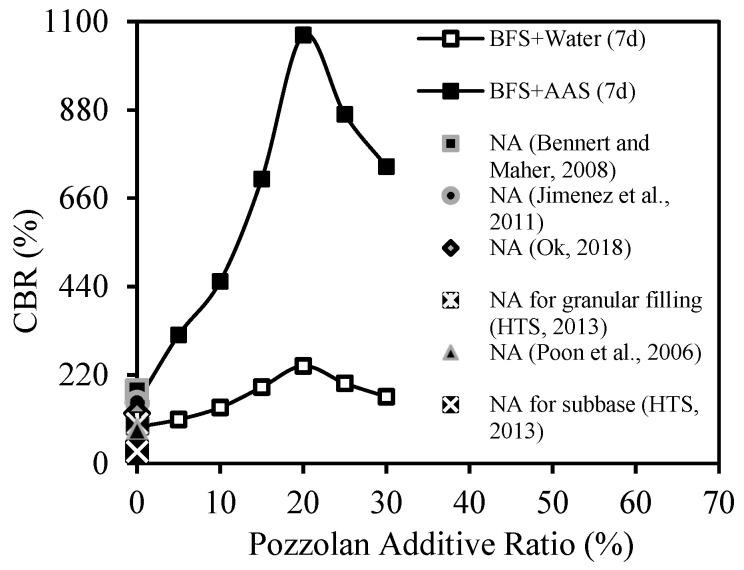
A comparison of the results of tests with those of literature [56,57,58,59,60] for CBR tests.

The results of CBR tests on samples cured for 7 days were compared with the CBR values suggested by some of the studies in the literature (see Figure 22 and Table 9).

In the literature studies considered, natural aggregate (NA) was used. The CBR values of the samples with a BFC additive and water closely approached the values in the literature. However, the CBR values of the samples with BFC+AAS additives exceeded the values in the literature by a significant margin, even at a BFC concentration of 5%.

**Figure 23:**
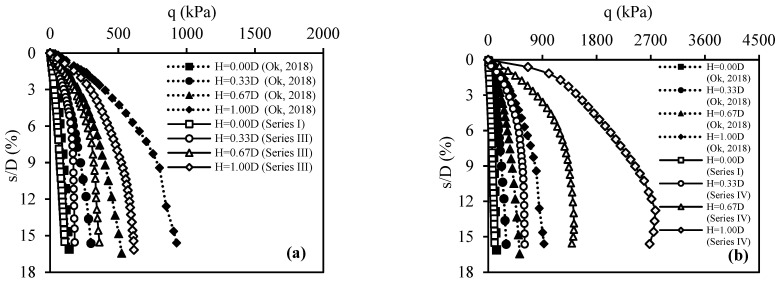
A comparison of the results of plate-loading tests with those of the literature [58]: (**a**) Series III; (**b**) Series IV.

The model plate-loading tests’ results of the CDW fill layer and the CDW fill layer with BFS+AAS were compared with those of the fill layer created with natural aggregates reported by Ok [58] to interpret the usability of CDW in the fill layer. The CBR and undrained shear strength values of the weak soil used by Ok [58] were 1% and 25 kPa, respectively. Moreover, the CBR value of the natural granular soil used in the fill layer was 125.16%, and this granular soil was classified as GW, according to UCSC. In addition, the dimensions of the rigid test tank and model foundation Ok [58] used are the same as those of this study. A comparison of the results of Series III and Series IV in this study with those of the model plate-loading tests conducted by Ok [58] is shown in Figure 23. The plate-loading test results for the weak soil in this study were similar to those presented by Ok [58]. As a result, it was concluded that the performance of fill layers in the two studies can be compared. Figure 23a shows that the performance of the CDW fill layer was inferior to that of the fill layer containing natural aggregates for all fill layer thicknesses. Additionally, it was determined that the CDW fill layer should be made thicker, approximately 0.33 D, to achieve the same performance as the fill layer created from natural aggregates. Based on Figure 23b, the performance of the CDW fill layer with BFS-AAS was relatively better than that of the fill layer with natural aggregates. Additionally, the 1.00 D thick fill layer with natural aggregates showed similar performance to the 0.33 D thick CDW fill layer with BFS-AAS.

## 5. Discussion

In the study by Ok [58], which confirms this study, CDW can be used as a filling material, and improving the properties of CDW can even be used as a strategy in filling constructions that require higher strength. As stated in the study by Sosahab et al. [61], high strength can be achieved in CDW treated with BFS additive due to the formation of binder gels that bind CDW particles together and form a hardened mass. This effect is particularly noticeable with the usage of the AAS. In addition, the amount of binder gels in the CDW mixture increased with increasing curing time, resulting in increased strength values. However, while the BFS additive increases the strength up to a certain ratio, it has no strength-increasing effect after this ratio. The biggest reason for this is the unreacted BFS.

As mentioned in the study by Figiela et al. [62], CDW can be used as a material in geopolymer production. Both this study and literature studies have shown that it is possible to produce geopolymer composites using the materials that make up CDW. In fact, such studies show the potential for useful materials composed of industrial by-products and CDW that provide environmental benefits. The usage of such materials in road base/subbase structures and foundation filling will help to utilize recycled materials, conserve natural resources, and reduce the carbon footprint. In addition, as shown in this study, CDW can be used without any decomposition to produce high-strength filling without expending any decomposition energy [21,23].

Furthermore, as suggested by Figiela et al. [62], the SEM-EDS analyses performed in this study provided useful information about the mineralogy and microstructure of the geopolymers produced by CDW-BFS-AAS and confirmed the CBR experiments and demonstrated the compact structure of the material produced by CDW-BFS-AAS. Also, the results of the EDS analysis showed that a typical geopolymer was produced by CDW-BFS-AAS. Moreover, as found in the study by Hamed and Demiröz [63], calcium silicate hydrate (C–S–H) and sodium aluminosilicate hydrate (N-A-S-H) gels, which are products of the geopolymerization reaction, were determined in the SEM-EDS analysis in this study.

Plate-loading tests can be used to determine the strength of geopolymer fillings [64]. In fact, one of the best methods in cases where a geopolymer filling is constructed on weak soil is the plate-loading test. In this study, this situation was seen more clearly when CBR and model plate-loading tests were compared. Because the stress on the geopolymer filling also significantly affects the weak soil, they must be evaluated together.

It was seen in the literature that geotextile can increase the bearing capacity of CDW fillings [65,66]. Similarly, in this study, it was observed that the geotextile used in geopolymer fillings increased the bearing capacity. It was also observed that the geotextile fulfills the separation function remarkably well. This is actually an important point, because there may be substances harmful to nature among the products entering the geopolymer reaction. These substances can be prevented from reaching nature by being absorbed/prevented by geotextile.

The results of the study showed that geopolymer filling and/or geotextile increased the bearing capacity of weak soil. However, this increase alone is not sufficient to recommend the usage of these methods as soil improvement. Therefore, it is necessary to consider these methods in all their aspects. Firstly, if the performance improvement aspect is taken into account, these methods increase the bearing capacity of the soil, resulting in a longer lifespan of the structure. This can result in less maintenance and repair costs. In addition, due to the fact that the geotextile absorbs the stresses and the geopolymer filling acts as a rigid plate, the total and differential settlement is reduced, and the stability of the structure is increased. This increases the functional and aesthetic life of the structure. Considering the economic point of view, it is obvious that geotextile reinforcement or geopolymer filling can bring an additional cost to the project. However, this cost should be compared with the situation without ground improvement and with other ground improvement methods. If geotextile reinforcement or geopolymer filling is not used, a thicker foundation layer, excavation filling works, or deeper excavations may be required. These costs may be higher than the construction of geotextile reinforcement or geopolymer filling. Since these methods increase the bearing capacity and reduce settlement, they can compensate the initial investment cost by making long-term savings. Therefore, this point should be analyzed. Finally, the usage of these methods can reduce material transport and environmental impact by reducing the need for natural filling material. This is very important for sustainability.

## 6. Conclusions

This study examines the performance of different fill layers made with CDW through laboratory and model plate-loading tests, as well as SEM analyses. It also explores ways to improve the performance of the CDW fill layer by using geotextiles and additive materials like BFS and AAS. The findings from this study are summarized below.

The compaction parameters (γ_dmax_ and ω_opt_) obtained from the compaction test on BFS and water-added CDW samples were similar to the compaction parameters (γ_dmax_ and ω_optAAS_) obtained from the compaction test on BFS- and AAS-added CDW samples. It was predicted that the possible lubricating effect of AAS reduced the friction between the CDW grains. Also, it was estimated that the increase in optimum liquid content values and decrease in γ_dmax_ values as the BFS ratio increased was due to the high water-absorption potential of BFS due to its high CaO content and the finer grain and lower mass of BFS compared to CDW.

After conducting laboratory tests, it was found that the samples containing 20% BFS+AAS additive exhibited the highest strength values for both 7-day and 28-day cure times. However, when an excessive amount of BFS was added, some BFS grains remained unreacted, as supported by SEM images. The unreacted BFS grains increased the fine content in the CDW sample, leading to a decrease in strength rather than contributing to it.

It was observed that increasing the curing time led to higher CBR values, but lower strain values when failure occurred or the limit displacement was exceeded, making the sample more ductile.

SEM images of the CDW sample with water added and cured for 28 days showed that CSH and Ca(OH)_2_ phases had formed in the microstructure. This circumstance demonstrated that a small amount of unreacted cement particles had reacted, providing a slight increase in strength. On the other hand, SEM images of the CDW sample with AAS added and cured for 28 days showed that ettringite crystals had formed, providing a more rigid sample than the CDW sample with water added and cured for 28 days. Therefore, this situation indicated that the AAS had reacted.

SEM images of the CDW sample with 20% BFS added showed the formation of a dense aluminosilicate gel and Ca(OH)_2_ in the matrix. However, a small number of unreacted BFS grains were also observed in the microstructure. On the other hand, SEM images of the CDW sample with 20% BFS and AAS added demonstrated dense aluminosilicate gels formed due to the geopolymerization reaction of the BFS with AAS, which filled the voids in the CDW sample.

According to model plate-loading tests’ results, the geotextile placed as a separator at the filler–weak soil interface had a negligible contribution to the bearing capacity. However, it effectively fulfilled its separator function. Therefore, it was suggested that placing the geosynthetics fill layer between the subgrade would help preserve the integrity of the fill layer.

The results of model plate-loading tests demonstrated that adding 20% BFS+AAS to CDW significantly increases the fill layer’s bearing capacity. For fill layer thicknesses of H = 0.33 D, 0.67 D, and 1.00 D, the ultimate bearing capacity increased by about 3, 4, and 4.5 times, respectively, compared to the unreinforced CDW fill layer. The degree of improvement decreased as the displacement ratio (s/D) decreased but increased as the fill thickness increased.

The CDW fill layer should be increased in thickness by approximately 0.33 D in order to achieve equivalent performance to the fill layer comprising natural aggregates. However, the ultimate bearing capacity of the CDW fill layer with 20% BFS and AAS added was greater than that of the fill layer made by natural aggregates, approximately 2–3 times. Therefore, a CDW fill layer can be constructed with 20% BFC and AAS added, allowing for a reduction in thickness by half compared to a fill layer composed solely of natural aggregates.

It has been concluded that CDW could serve as an alternative fill material to natural aggregates. Furthermore, it has been determined that adding BFC and AAS enhances CDW’s performance. Utilizing CDW and BFC in the fill layer instead of depositing them in landfills could have environmental and economic benefits, especially in the aftermath of disasters such as the earthquake in Türkiye on 6 February 2023, which resulted in a high CDW volume.

## Figures and Tables

**Table 1:** The types and proportions of materials contained in the CDW.

Type of Material	Unit	Value
Aggregate without binder, aggregate with binder, etc.	(%)	52.11
Concrete products, mortar, etc.	(%)	36.21
Wall units with calcium silicate, brick, etc.	(%)	11.14
Other materials such as non-floating wood plastic, metals, rubber, plaster, etc.	(%)	0.44
Glass and glass-like materials	(%)	0.09
Floating particles	(cm^3^/kg)	0.06

**Table 2:** The properties of the CDW.

Properties	Units	Value	Standard
Coefficient of uniformity (C_u_)	-	20.00	[37]
Coefficient of curvature (C_c_)	-	1.12	[37]
Soil classification	-	SW	[38]
Flakiness index	%	11.14	[39]
Specific gravity for fine particles (G_s_)	-	2.621	[40]
Water absorption for fine particles	%	6.91	[40]
Specific gravity for coarse particles (G_s_)	-	2.603	[41]
Water absorption for coarse particles	%	3.94	[41]
Los Angeles abrasion loss	%	32.38	[42]
Maximum dry unit weight (γ_dmaks_)	kN/m^3^	19.79	[43]
Optimum water content (ω_opt_)	%	11.25	[43]
California bearing ratio (CBR)	%	81.28	[44]

**Table 3:** The properties of the fine-grained soil.

Properties	Units	Values	Standard
Soil classification	-	MH	[38]
CBR (at ω_opt_)	%	20.61	[44]
CBR (at ω = 46%)	%	2.19	[44]
Specific gravity (G_s_)	-	2.61	[45]
Liquid limit (ω_L_)	%	58.08	[46]
Plastic limit (ω_P_)	%	36.32	[46]
Plasticity index (ω_PI_)	%	21.76	[46]
Maximum dry unit weight (γ_dmaks_)	kN/m^3^	14.80	[47]
Optimum water content (ω_opt_)	%	28.25	[47]
Undrained shear strength (c_u_) (at ω_opt_)	kN/m^2^	204.36	[48]
Undrained shear strength (c_u_) (at ω = 46%)	kN/m^2^	26.05	[48]

**Table 4:** The chemical composition of BFS.

**Compounds**	SiO_2_	Al_2_O_3_	Fe_2_O_3_	CaO	MgO	SO_3_	S^−2^	Na_2_O	K_2_O	TiO_2_	Mn_2_O_3_	Cl^−^
**Unit**	%											
**Value**	32.47	9.94	1.25	32.45	9.31	0.82	0.33	0.31	0.85	1.16	3.51	0.015

**Table 5:** The properties of NaOH.

Properties	Molecular Formula	Molecular Mass	Color	pH	Relative Density
**Unit**	-	g/mol	-	-	kN/m^3^
**Value**	NaOH	40.0	White	13–14	21.20

**Table 6:** The properties of the geotextile.

Properties	Units	Values
Type	-	Non-woven
Raw material	-	Polypropylene
Unit weight	g/cm^2^	250
Opening size	mm	0.12
Thickness	mm	1.50
Elongation	%	50
Static puncture resistance	N	2500
Tensile strength	kN/m	13/15
Permeability	m/s	0.06
Dynamic perforation	mm	20
UV resistance	%	70

**Table 7:** The model plate-loading test series.

Test Series	Test Number	Presence of the CDW	Presence of the BFS	Presence of the AAS	Presence of the Geotextile	Foundation Diameter (D) (mm)	Height of the Test Section (mm)
Series I	1	✘	✘	✘	✘	150	350
Series II	2	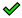	✘	✘	✘		Series I + 50
	3						Series I + 100
	4						Series I + 150
Series III	5	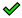	✘	✘	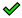		Series I + 50
	6						Series I + 100
	7						Series I + 150
Series IV	8	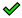	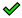	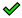	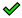		Series I + 50
	9						Series I + 100
	10						Series I + 150

Note: “
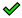
” indicates presence and “✘” indicates non-presence.

**Table 8:** The elemental composition of the test specimens obtained from EDS.

Element	BFS Mass (%)	CDW Mass (%)	CDW+AAS Mass (%)	CDW+20%BFS Mass (%)	CDW+20%BFS+AAS Mass (%)
Ca	6.91	8.18	4.87	4.77	10.05
Si	7.45	3.10	3.84	3.19	6.06
C	3.45	6.14	5.86	9.54	7.01
Al	2.52	0.49	1.32	0.79	1.77
Mg	2.26	0.81	1.22	0.75	1.58
Mn	0.35	x	x	0.32	0.47
S	0.58	x	x	x	0.56
Ti	0.25	x	0.11	0.18	0.21
Fe	0.16	1.11	1.06	0.33	x
K	0.12	x	0.28	0.10	0.06
Na	0.16	x	1.91	0.08	2.58
O	75.80	80.19	79.54	79.94	69.65

Note: “x” means that the element is not present.

**Table 9:** An ascending list of the CBR values of the test specimens in this study and the literature.

Sample Name	CBR (%)	Sample Name	CBR (%)
NA for subbase [56]	30.00	NA [60]	182.00
NA [57]	83.00	This study (15%BFS-7d)	189.90
This study (CDW-7d)	90.59	This study (25%BFS-7d)	199.46
NA for granular filling [56]	100.00	This study (20%BFS-7d)	242.99
This study (5%BFS-7d)	109.28	This study (5%BFS-AAS-7d)	319.98
NA [58]	125.16	This study (10%BFS-AAS-7d)	453.62
This study (10%BFS-7d)	139.55	This study (15%BFS-AAS-7d)	708.25
This study (CDW-AAS-7d)	151.22	This study (30%BFS-AAS-7d)	738.92
NA [59]	152.00	This study (25%BFS-AAS-7d)	868.89
This study (30%BFS-7d)	166.00	This study (20%BFS-AAS-7d)	1066.87

## Data Availability

The original contributions presented in this study are included in the article. Further inquiries can be directed to the corresponding author.
